# Human Pancreatic Cancer-Associated Stellate Cells Remain Activated after *in vivo* Chemoradiation

**DOI:** 10.3389/fonc.2014.00102

**Published:** 2014-05-09

**Authors:** M. Carla Cabrera, Estifanos Tilahun, Rebecca Nakles, Edgar S. Diaz-Cruz, Aline Charabaty, Simeng Suy, Patrick Jackson, Lisa Ley, Rebecca Slack, Reena Jha, Sean P. Collins, Nadim Haddad, Bhaskar V. S. Kallakury, Timm Schroeder, Michael J. Pishvaian, Priscilla A. Furth

**Affiliations:** ^1^National Cancer Informatics Program, National Cancer Institute, National Institutes of Health, Bethesda, MD, USA; ^2^Lombardi Comprehensive Cancer Center, Department of Oncology, Georgetown University, Washington, DC, USA; ^3^Department of Pharmaceutical Sciences, College of Pharmacy, Belmont University, Nashville, TN, USA; ^4^Department of Gastroenterology, Georgetown University, Washington, DC, USA; ^5^Department of Radiation Medicine, Georgetown University, Washington, DC, USA; ^6^Department of Surgery, Georgetown University, Washington, DC, USA; ^7^Department of Biostatistics, University of Texas MD Anderson Cancer Center, Houston, TX, USA; ^8^Department of Radiology, Georgetown University, Washington, DC, USA; ^9^Department of Pathology, Georgetown University, Washington, DC, USA; ^10^Helmholtz Zentrum München – German Research Center for Environmental Health, Research Unit Stem Cell Dynamics, Neuherberg, Germany; ^11^Department of Biosystems Science and Engineering, ETH Zurich, Basel, Switzerland; ^12^Division of Hematology/Oncology, Department of Medicine, Georgetown University, Washington, DC, USA

**Keywords:** pancreatic cancer, stellate cells, chemotherapy, radiation, gemcitabine, PDAC

## Abstract

Pancreatic ductal adenocarcinoma (PDAC) is characterized by an extensive fibrotic reaction or desmoplasia and complex involvement of the surrounding tumor microenvironment. Pancreatic stellate cells are a key mediator of the pancreatic matrix and they promote progression and invasion of pancreatic cancer by increasing cell proliferation and offering protection against therapeutic interventions. Our study utilizes human tumor-derived pancreatic stellate cells (HTPSCs) isolated from fine needle aspirates of pancreatic cancer tissue from patients with locally advanced, unresectable pancreatic adenocarcinoma before and after treatment with full-dose gemcitabine plus concurrent hypo-fractionated stereotactic radiosurgery. We show that HTPSCs survive *in vivo* chemotherapy and radiotherapy treatment and display a more activated phenotype post-therapy. These data support the idea that stellate cells play an essential role in supporting and promoting pancreatic cancer and further research is needed to develop novel treatments targeting the pancreatic tumor microenvironment.

## Introduction

Pancreatic ductal adenocarcinoma (PDAC) is a lethal malignancy with poor prognosis (1). It is characterized by its rapid progression, early local invasion and metastasis, and poor response to chemotherapy and radiotherapy (1–3). An extensive fibrotic reaction or desmoplasia (4) and complex involvement of the surrounding tumor microenvironment are common defining factors of PDAC (5). The stromal compartment of the pancreas is composed of extracellular matrix (ECM) proteins, pancreatic stellate cells (PSCs), and immune cells. The exact mechanisms of involvement of this tumor matrix are still being elucidated. However, recent findings show that interactions between pancreatic cancer cells (PCCs), ECM proteins, and growth factors secreted by stromal components activate intracellular signals that protect cancer cells from apoptosis (4). Furthermore, PSCs stimulate the growth of PCCs by increasing tumor cell proliferation, promoting epithelial–mesenchymal transition, enhancing metastasis, and providing radioprotection to tumor cells (6–9).

Pancreatic stellate cells are myofibroblast-like cells found in the stromal compartment of the pancreas in interlobular areas and in interacinar regions (10). They have a flattened angular appearance with elongated cytoplasmic processes on phase-contrast microscopy and are characterized by the presence of numerous lipid droplets in the cytoplasm containing auto-fluorescent vitamin A stored as retinyl palmitate. During pancreatic injury of inflammation, PSCs transform from a quiescent state into an activated, myofibroblast-type phase. Studies in rodents and humans have identified several cytokines, growth factors, and signaling molecules as regulators of PSC activation (11–13). Morphologic, molecular, and behavioral features and markers of PSC activation have been identified as: increased cell migration and contraction, enlarged nucleus, increased proliferation, and expression of intermediate filament proteins vimentin, desmin, and glial fibrillary acidic protein (GFAP) (10–12, 14–17). These markers, in addition to the presence of intracellular lipid droplets, aid in distinguishing activated PSCs from normal fibroblasts.

Emerging studies suggest that ablation of cancer stem cells (CSCs) is of critical significance for effective treatment cancer (18–20). Recent data suggest that PSCs may enhance the cancer stem-like phenotype in PCCs (21–23). Hamada and colleagues show that indirect co-culture of PCCs with PSCs enhanced the spheroid-forming ability of cancer cells and induced the expression of cancer stem cell-related genes ABCG2, Nestin, and LIN28 (21). In a complimentary study, Lonardo and group demonstrate that PSCs are an important component of the tumor stroma for creating a paracrine niche for pancreatic CSCs. In their model, PSC-derived Nodal/Activin drove self-renewal and invasiveness of pancreatic CSCs (22). These studies suggest that targeting PSCs and other tumor microenvironment components can assist in disrupting the cancer stem cell niche and should be considered as a part of cancer stem cell treatment strategies.

Two reports have described the protective effect that PSCs impart against chemotherapy and radiation on PCCs (6, 8). However, *in vitro* studies on human tumor-derived pancreatic stellate cells (HTPSC) are limited (24) and little is known about the direct effect that chemotherapy and radiation have on human PSCs (24, 25). Our study shows that human cancer-associated PSCs can be isolated from fine needle aspirates (FNAs) of pancreatic cancer tissue from patients with locally advanced, unresectable pancreatic adenocarcinoma before and after treatment with full-dose gemcitabine plus concurrent hypo-fractionated stereotactic radiosurgery (SRS) and maintained in primary culture for studies of cell behavior. We isolated paired samples of HTPSCs before and after treatment from FNAs of *in situ* tumors in patients enrolled in a study assessing the utility of sequential tumor sampling for assessing therapeutic response. Cells were placed into primary cell culture and HTPSCs were identified by a combination of *in situ* techniques and morphology. A novel single-cell tracking software was utilized to characterize the behavior of the cultured HTPSCs. Cell activation (motility, locomotion, membrane expansion/contraction) was compared before and after combination chemotherapy/radiation. HTPSCs showed evidence of increased activation post-therapy with increased motility and locomotion and greater ratios of membrane expansion and contraction as compared to HTPSCs isolated from the same patient before treatment. The findings are compatible with the notion that current therapies targeting PCCs may not inactivate PSCs. Since it is possible that activated PSCs contribute to survival of PCCs being targeted by the chemotherapy and radiation, treatments may need to be broadened to include targeting of this cell type as well (23, 26–28). Further studies elucidating stellate-specific markers for inactivation and their therapeutic targets may lead to novel combination therapies directed to this significant component of the pancreatic adenocarcinoma microenvironment.

## Materials and Methods

### Ethic statement

This study was approved by the Georgetown University Oncology Institutional Review Board of Washington, DC, USA, and followed the Ethical Principles for Medical Research Involving Human Subjects from the Declaration of Helsinki. Written informed consent was obtained from subjects.

### Clinical trial

**Figure 1:**
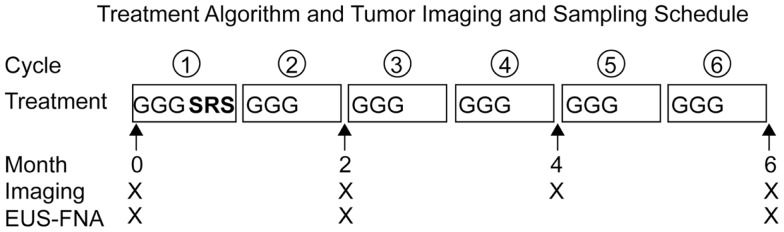
**Treatment algorithm and tumor imaging and sampling schedule**. Patients with locally advanced, unresectable pancreatic adenocarcinoma received full-dose gemcitabine (G) plus concurrent hypo-fractionated stereotactic radiosurgery (SRS). Stereotactic radiosurgery was administered on the off week of the first cycle of gemcitabine. Restaging imaging was performed every 2 months. Endoscopic ultrasound with fine needle aspiration (EUS-FNA) with fiducial placement was performed prior to therapy and serial EUS-FNAs were performed after 2 and 6 months of therapy.

Patients with previously untreated locally advanced, unresectable pancreatic cancer were enrolled in a pilot study designed to demonstrate the feasibility and safety of administering hypo-fractionated SRS concurrently with full-dose gemcitabine (G). In an effort to decrease late duodenal toxicity, we examined the use of fractionated SBRT with full-dose gemcitabine (29). Patients received gemcitabine (1000 mg/m^2^) on days 1, 8, and 15 of every 28-day cycle, for up to 6 months. Patients were also treated with 5 Gy of SRS daily over five consecutive days (typically days 22–26) of Cycle 1 only. Patients also underwent serial endoscopic ultrasound-guided fine needle aspiration (EUS-FNA) of pancreatic masses prior to therapy, after 2 months, and after 6 months (Figure 1).

### Primary human pancreatic cell culture protocol

Primary cultures of HTPSCs were generated from EUS-FNA aspirates obtained from patients with locally advanced, unresectable pancreatic adenocarcinoma before treatment (Pre) and at 6 months following treatment with gemcitabine (G) and SRS (Post) (Figure 1). Immediately upon collection, FNAs were placed in DMEM (high glucose) (Invitrogen, Carlsbad, CA, USA) with 1% fetal bovine serum (FBS), 10 mL/L of antibiotic–antimycotic (Invitrogen, Carlsbad, CA, USA) and moved to a laminar flow tissue culture hood in the laboratory. All subsequent manipulations were performed using standard sterile tissue culture techniques. After collection, 1 mL of dispase and 2 mL of Trypsin–EDTA were added to the media and the tubes were incubated for 30 min at 37°C with occasional vortexing. After incubation, the tissue was further dissociated by triturating with a sterile pipette. The pancreatic tumor cell suspensions were then subjected to gravity centrifugation for 5 min at 1800 rpm. The pellets were resuspended in 5 mL of FCM Lysing Solution (Santa Cruz Biotechnology, Santa Cruz, CA, USA) to lyse red blood cells and incubated at room temperature for 5 min with occasional trituration with sterile pipette. The pancreatic cell suspensions were again subjected to gravity centrifugation for 5 min at 1800 rpm. After the supernatant was removed, the pellets were resuspended in 2 mL of complete media containing DMEM (high glucose), 10% FBS, 10 ng/mL epidermal growth factor (EGF), 100 U of human insulin (per 500 mL of medium), and 10 mL/L of antibiotic–antimycotic. The cultures were plated in a single well of a six-well tissue culture treated plate and placed securely on humidified incubation chamber of inverted Nikon Eclipse TE300/PerkinElmer Spinning Disc Confocal microscope system (Waltham, MA, USA) at 37°C with 5% CO_2_. Cells were allowed to attach for 24 h, wells were gently washed of debris with media, and replaced with fresh complete media previously equilibrated for CO_2_ in the incubator and warmed at 37°C.

### Cell imaging

Images were obtained with inverted Nikon Eclipse TE300/PerkinElmer Spinning Disc Confocal microscope system (Waltham, MA, USA) with humidified incubation chamber using 10× objective lens in phase-contrast mode and Volocity software (v 5.3.1, PerkinElmer, Waltham, MA, USA) as previously described (30). Briefly, three to five sets of 812 μm × 875 μm contiguous fields were selected in a square (5 × 5) configuration in the middle of each well depending on cellular location. A focus map was created for each point and time-lapse image acquisition was set in 15-min intervals. Cells were imaged for 3–7 days. One to two milliliters of complete media were added daily.

### Immunohistochemistry

Immediately after time-lapse imaging, cells were prepared in the respective well of a six-well plate and stained by immunofluorescence with vimentin 1:50 (sc-7557; Santa Cruz Biotechnology, Santa Cruz, CA, USA) and fluorescently labeled secondary antibody FITC anti-goat (sc-2024; Santa Cruz Biotechnology, Santa Cruz, CA, USA). Sections (5 mm thick) of formalin-fixed, paraffin-embedded pancreatic adenocarcinoma FNAs were prepared and stained by immunohistochemical staining with GFAP (GA5) 1:50 (3670S; Cell Signaling, Danvers, MA, USA). Digital photographs taken with Nikon Eclipse E800M microscope with DMX1200 software; Nikon Instruments, Inc. (Melville, NY, USA) and Olympus IX71 with Olympus DP Capture Software (Center Valley, PA, USA).

For immunohistochemistry, heat induced epitope retrieval (HIER) was performed by immersing the tissue sections at 98°C for 20 min in 10 mM citrate buffer (pH 6.0) with 0.05% Tween. Immunohistochemical staining was performed using a horseradish peroxidase labeled polymer (K4001; Dako, Carpinteria, CA, USA) according to manufacturer’s instructions. Briefly, slides were treated with 3% hydrogen peroxide and 10% normal goat serum for 10 min each, and exposed to primary antibody for GFAP (1:50, 3670; Cell Signaling, Danvers, MA, USA) for 1 h at room temperature. Slides were exposed to anti-mouse labeled HRP for 30 min and DAB chromagen (Dako, Carpinteria, CA, USA) for 5 min. Slides were counterstained with Hematoxylin (Harris Modified Hematoxylin; Fisher, Waltham, MA, USA) at a 1:17 dilution for 2 min at room temperature, blued in 1% ammonium hydroxide for 1 min at room temperature, dehydrated, and mounted with Acrymount (McKinney, TX, USA). Sections with the omitted primary antibody were used as negative controls.

For immunofluorescence, cells in each well of a six-well plate were fixed for 15 min in 4% formaldehyde, rinsed with PBS, blocked for 20 min with 10% normal blocking serum, and incubated with primary antibody for vimentin (1:50, sc-7557; Santa Cruz Biotechnology, Santa Cruz, CA, USA) for 60 min at 37°C. After washing, secondary antibody was applied for 45 min in a dark chamber. After a final washing, wells were covered with anti-fade reagent (#9071; Cell Signaling, Danvers, MA, USA) with DAPI (#8961; Cell Signaling, Danvers, MA, USA).

### Cell tracking

After 3–7 days of time-lapse image acquisition, Timm’s Tracking Tool (TTT) (31, 32) was used to track individual cell migration as previously described (30). Briefly, time-lapse images for each point were loaded into TTT; individual human tumor-derived pancreatic stellate cells (HTPSCs) were identified on the screen by morphology (a flattened angular appearance and long cytoplasmic processes giving them a typical “stellate” appearance) and the presence of multiple cytoplasmic lipid-droplet deposits. Each cell’s trajectory through the time-lapse images was recorded in TTT by following each cell’s nucleus.

### Cell motility, speed, and membrane expansion/contraction measurements

**Figure 2:**
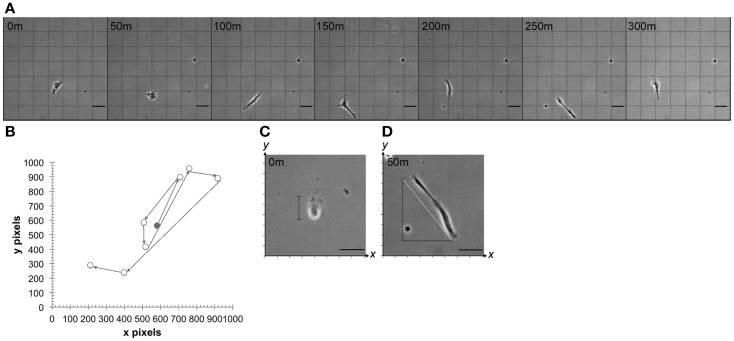
**Cell motility, speed, and membrane expansion/contraction parameters**. **(A)** Time-lapse series of images showing multidirectional migration of a representative human tumor-derived pancreatic stellate cell at 50-min intervals. **(B)** Representative cell path used to determine total distance traveled by tracking individual cell nucleus in time-lapse video-microscopy using TTT software. Circles represent the cell nucleus at observed points when change of direction took place. Shaded circle represents nucleus at time 0. Cell expansion and contraction were measured by **(C)** calculating expanded cell diameter on *x*,*y* or **(D)** by calculating the hypotenuse of the triangle (white line) representative of expanded cell after obtaining *x*,*y* coordinates (m = minutes). Minutes represent elapsed time after first observation *in vitro*. Scale bar: 50 μm.

As described above, time-lapse images for each point were loaded into TTT and individual HTPSCs were identified on the screen by morphology and cytoplasmic lipid-droplet deposits. Total cell distance traveled in the *x*,*y* axis was recorded via TTT tracking (Figure 2A) and cell speed was calculated by *s* = *d*/*t* where *d* is distance traveled and *t* is total time of travel. Additionally, each change of direction event was recorded manually by graphing the *x,y* coordinates of distance traveled and directional change (Figure 2B). For each cell, the base diameter was measured in pixels by recording the *x,y* coordinates of the cell boundary. The total pixels were converted to micrometers (0.73 μm/pixel). Cells were observed in the time-lapse images and membrane expansion and contraction events were noted whenever the cell membrane expanded by greater than 50% of total body length. During each membrane expansion, the *x*,*y* coordinates of the cell boundary were recorded at the cell’s longest points (Figure 2C). If the cell was diagonally positioned, the expansion length was calculated using the Pythagorean theorem *a*^2^ + *b*^2^ = *c*^2^, where *c* is cell diameter (Figure 2D). The expansion and contraction ratio was calculated by *x* = *b*/*e* where *b* is the base cell diameter and *e* is the expanded cell diameter.

### Statistical analysis

Differences and variance (σ) in mean cell speed, total distance traveled, and expansion and contraction ratios were compared with two-way ANOVA (GraphPad Prism version 6 for Mac, GraphPad Software, La Jolla, CA, USA). Significance was assigned at *p* < 0.05; *n* = 3 patients per cohort.

## Results

### Human tumor-derived pancreatic stellate cells can be isolated from fine needle aspirates and maintained in culture

**Figure 3:**
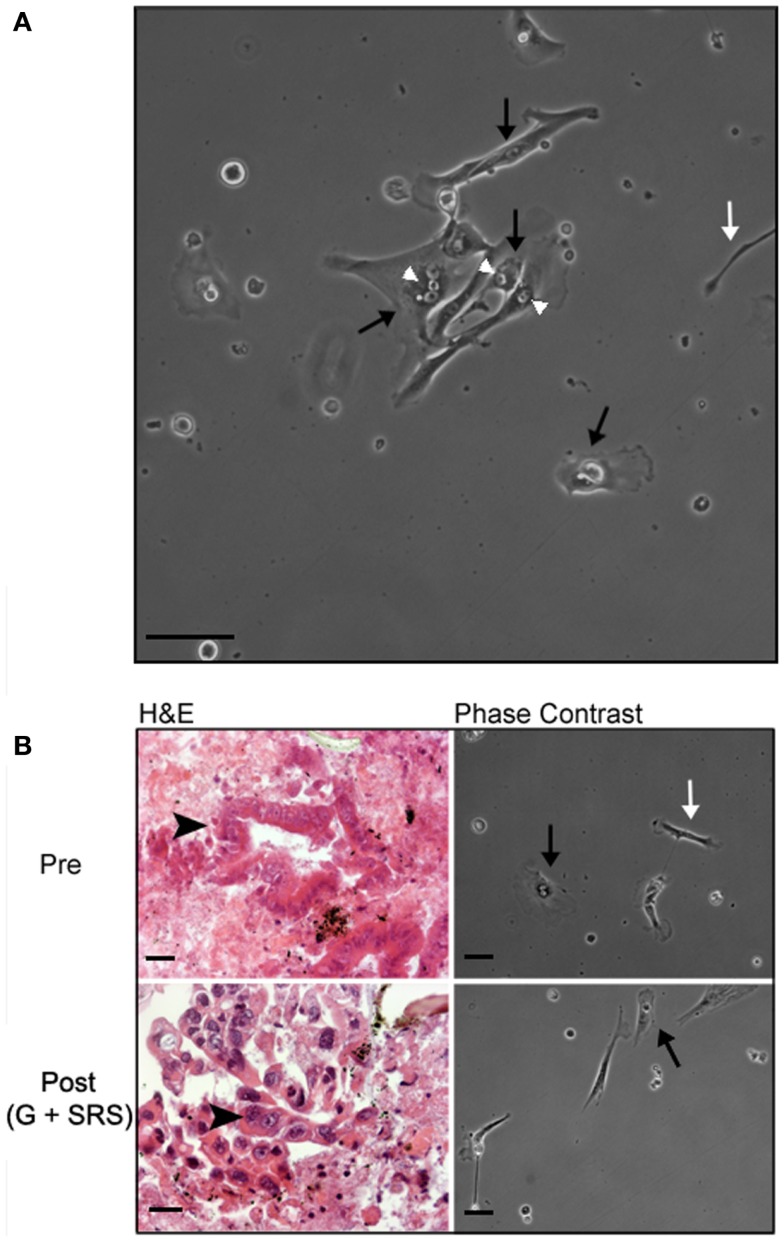
**Histology and morphology of human tumor-derived pancreatic stellate cells**. **(A)** Representative phase-contrast image of human tumor-derived pancreatic stellate cells in monolayer after 72 h in culture. EUS-FNAs were placed in standard cell culture conditions, resulting in the outgrowth of myofibroblast-like cells. These stellate cells are characterized by a flattened angular appearance, the presence of cytoplasmic lipid droplets, long cytoplasmic processes giving them a typical “stellate” appearance, and cytoplasmic expansion and contraction behavior. **(B)** Representative hematoxylin/eosin (H&E) stained section and phase-contrast bright field images from time-lapse movies of cell cultures from patients with locally advanced, unresectable pancreatic adenocarcinoma before treatment (pre) and at 6 months following treatment with gemcitabine (G) and stereotactic radiosurgery (SRS) (post). White arrowheads point to cytoplasmic lipid droplets; black arrows point to lipid-droplet containing pancreatic stellate cells; white arrows point to other non-stellate cells in culture; black arrowheads point to pancreatic ductal adenocarcinoma cells. Scale bar: 50 μm.

Human tumor-derived pancreatic stellate cells were identifiable by morphology in primary culture after 16–24 h of incubation and formed small colonies after 48–72 h (Figure 3A). HTPSCs were the predominant cell type identified during culture; the heterogeneous culture also included epithelial cells and non-stellate fibroblasts. HTPSCs were initially characterized by a flattened angular appearance, the presence of multiple cytoplasmic lipid droplets, long cytoplasmic processes giving them a typical “stellate” appearance, and cytoplasmic expansion and contraction behavior. HTPSC isolation correlated with diagnostically adequate EUA-FNA samples collected from patients with locally advanced, unresectable pancreatic adenocarcinoma before treatment (pre) and at 6 months following treatment with gemcitabine (G) and SRS (post) (Figure 3B).

### Human tumor-derived pancreatic stellate cells stain positive for GFAP and vimentin

**Figure 4:**
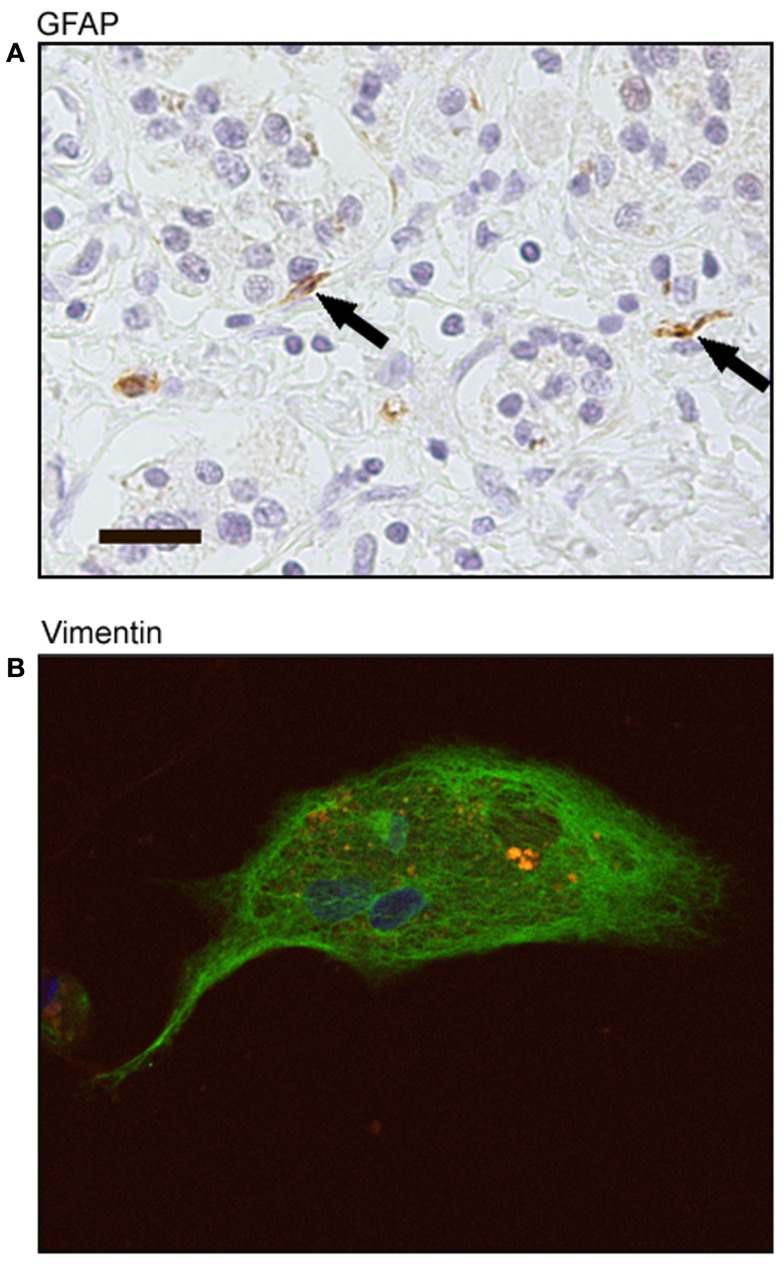
**Immunohistochemical staining of human tumor-derived pancreatic stellate cells**. **(A)** Representative sections of pancreatic adenocarcinoma with positive staining of glial fibrillary acidic protein (GFAP) (black arrows). Scale bar: 20 μm. **(B)** Representative immunofluorescence staining of human tumor-derived pancreatic stellate cells with positive vimentin expression (FITC green) and auto-fluorescent cytoplasmic lipid droplets (orange). Nuclei counterstained with DAPI.

Pancreatic adenocarcinoma sections showed positive staining for GFAP localized to stellate-shaped cells and their cytoplasmic processes. These stellate-shaped cells were localized in the interacinar space with cytoplasmic processes extending around the base of adjacent acinar cells (Figure 4A). After 72 h in culture, cells stained positive for vimentin showing a fibrillar staining pattern in the cell cytoplasm and had the presence of auto-fluorescent lipid droplets (Figure 4B). These markers of stellate cells were correlated with the morphological characteristics we saw in culture of live cells: a flattened angular appearance, the presence of multiple cytoplasmic lipid droplets, long cytoplasmic processes, and cytoplasmic expansion and contraction behavior.

### Stellate cell behavior is altered in samples isolated from patients treated with full-dose gemcitabine plus concurrent hypo-fractionated SRS

**Figure 5:**
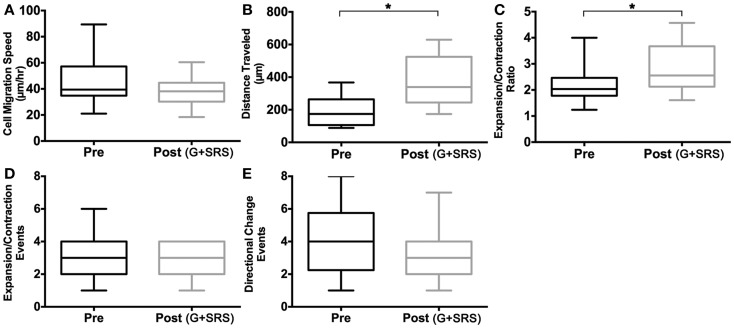
**Cell activation measurements**. Human tumor-derived pancreatic stellate cell migration was quantified and compared in cultures isolated from patients with locally advanced, unresectable pancreatic adenocarcinoma before treatment (Pre) and at 6 months following treatment with gemcitabine (G) and stereotactic radiosurgery (SRS) (Post). Box plots comparing **(A)** mean cell speed (micrometer per hour) show no significant rate of change of position when traveling in culture. **(B)** Total mean distance traveled (micrometer) is increased in human tumor-derived pancreatic stellate cells post-treatment, and **(C)** expansion and contraction ratio is higher in cells post-treatment. **(D)** Mean expansion and contraction events and **(E)** directional change events per cell showed no significant difference pre and post-treatment. Asterisks (*) indicate statistically significant changes. *p < 0.05, two-way ANOVA, *n* = 3 patients per cohort.

To define the parameters of individual cell motility, we measured cell migration speed, total distance traveled, and expansion/contraction ratios in HTPSCs isolated from FNAs of patients with locally advanced, unresectable pancreatic adenocarcinoma. Behaviors of individual cells isolated at baseline before treatment (Pre) were compared to cells isolated at 6 months following treatment with G + SRS (Post) (Figures 4 and 5). Although the speed of HTPSC migration was not significantly different in the pre-treatment versus post-treatment samples (Figure 5A), the variance of speed was greater in cells from the pre-treatment samples [21–90 μm/h (σ = 291)] compared to cells from the post-treatment samples [24–60 μm/h (σ = 99)] (ANOVA, *p* < 0.05) and the mean total distance traveled was 1.9-fold greater in cells from post-treatment samples (mean 198 SEM ± 21 pre; mean 370 SEM ± 33 post; ANOVA, *p* < 0.05). While pre-treatment cells traveled a maximum of 350 μm, post-treatment cells traveled up to 597 μm over the same time frame (Figure 5B). Membrane extension was measured by calculating expansion/contraction ratios. On average, cells pre-treatment extended 2× their body length compared to a 2.8× extension in cells post-treatment, a 1.3-fold increase (ANOVA, *p* < 0.05) (Figure 5C). The number of times these expansion/contraction events took place was not different between groups (Figure 5D).

## Discussion

The tumor microenvironment plays an important role in the initiation, progression, and invasion of PDAC (5–7, 22, 33–35). Stellate cells are one of the major mediators in the microenvironment of the pancreas and are responsible for contributing to the malignant phenotype of PCCs (6, 9). Furthermore, *in vivo* and *in vitro* studies have shown that activated PSCs play a critical role in the pathogenesis of pancreatic fibrosis (desmoplasia) and pancreatitis (15, 36, 37) (a risk factor for pancreatic cancer) (38). PSCs are activated early during pancreatic injury as a result of necrosis and inflammation. Once activated, PSCs can regulate ECM remodeling, contribute to inflammatory cytokine signaling, and provide the major source of collagen. This excessive accumulation of ECM proteins leads to fibrosis and contributes to pancreatitis (33, 34, 39).

The complexity of the pancreatic microenvironment and its involvement in PDAC suggest that members of this matrix may also mediate therapeutic response. Although radiation therapy inhibits cell proliferation and induces apoptosis in many tumor cells, radiation has been shown to enhance pancreatic fibrosis in PDAC (33). Additionally, data from *in vitro* and *in vivo* animal studies show that PSCs mediate radioprotection of PCCs (8) and impair tumor cell response to chemotherapy and radiation (6). Importantly, recent publications have elucidated a role of PSCs in enhancing the stem-like phenotype of pancreatic CSCs and in driving self-renewal and invasiveness of pancreatic CSCs (21, 22). The stromal component of PDAC, including PSCs has been shown to support and protect PCCs from the deleterious effects of anti-cancer agents (6, 8, 23, 40). When a stellate cell-targeting agent was used in combination with chemotherapy, tissue delivery of gemcitabine was enhanced and tissue metformin concentration was doubled. Notably, the combination of a stellate cell-targeting agent with gemcitabine and metformin was effective in tumors previously resistant to mTOR inhibition (23). These studies suggest that targeting the tumor microenvironment could increase susceptibility of CSCs to therapy and enhance the efficacy of current treatments of PDAC.

Our study is the first report to evaluate stellate cells isolated from FNAs of pancreatic tumors in patients. The restricted supply of fresh tumor tissue could be a limiting factor when studying HTPSCs; our study provides a method for utilizing tissue obtained from ultrasound-guided FNAs. Combining isolation of these cells with single-cell tracking allowed us to study the behavior of these cells in primary culture. This technique could be useful in assessing the effect of other therapies on behavior of HTPSCs.

Earlier studies examined the effect of gemcitabine and radiotherapy on PCCs using conditioned PSC media, irradiating cells in culture, or by administering treatment to mice (6, 8, 33). Conditioned medium from cultured PSCs stimulated pancreatic tumor cell proliferation, migration, invasion, and anchorage-independent growth in a dose-dependent manner (6). When irradiated PSCs were co-cultured with PCCs, PCCs had increased clonogenic survival when cocultured with PSCs (8). PSCs were exposed to gemcitabine in culture and were practically unaffected by the gradient doses of 5-fluorouracil or gemcitabine (33).

Our study is the first to investigate HTPSCs after administration of chemotherapy and radiation directly to humans with PDAC. Using a novel single-cell tracking technology, we were able to follow individual HTPSCs isolated from each patient and quantify global changes of behavior after treatment. Our findings revealed that HTPSCs survived treatment with gemcitabine (G) plus concurrent hypo-fractionated SRS and had altered behavior showing increased activation. HTPSCs isolated from patients after treatment with G + SRS traveled at the same speed as cells isolated before treatment; however, they were more motile, traveling 1.9 times further than untreated cells and had greater membrane expansion ratios. The combination of increased locomotion and membrane expansion in post-treatment cells could possibly represent greater traction and increased intracellular contractile force (41, 42). Cell migration and membrane expansion/contraction are two markers of stellate cell activation; (15) therefore, our data suggest that G + SRS increases HTPSCs activation that could contribute to the tumor microenvironment. Our studies further suggest that therapies to ablate stellate cells should be used in conjunction with standard chemotherapy and radiation to counteract the potential of these agents in activation PSCs.

While our current study focuses on HTPSCs, the pancreatic tumor microenvironment is a heterogeneous structure that also includes CSCs, adipocytes, immune cells, endothelial cells, and pericytes (28). The technique employed here focused on HTPSCs, but it would be optimal to be able to analyze the global behavior of the tumor microenvironment concurrently to determine cell interaction and activation.

In summary, our findings reveal that HTPSCs can be successfully isolated from serial samples collected through ultrasound-guided FNAs obtained from patients with locally advanced, unresectable pancreatic adenocarcinoma. These cells survived in tissues from patients orthotopically treated with gemcitabine and hypo-fractionated SRS and displayed an activated phenotype with increased cell migration and membrane expansion following treatment. The ability to isolate these cells from patients undergoing therapy and study their behavior in primary culture may help our understanding of the pathophysiological role of this cell type in pancreatic cancer and develop therapies that may be able to specifically target these cells.

## Author Contributions

Contributors have met all criteria required for authorship.

## Conflict of Interest Statement

The authors declare that the research was conducted in the absence of any commercial or financial relationships that could be construed as a potential conflict of interest.
